# A Hybrid Protection Scheme for the Gait Analysis in Early Dementia Recognition

**DOI:** 10.3390/s24010024

**Published:** 2023-12-19

**Authors:** Francesco Castro, Donato Impedovo, Giuseppe Pirlo

**Affiliations:** Department of Computer Science, University of Bari Aldo Moro, 70125 Bari, Italy; donato.impedovo@uniba.it (D.I.); giuseppe.pirlo@uniba.it (G.P.)

**Keywords:** security healthcare, gait analysis, neurodegenerative disease, random projection, homomorphic cryptography, cancelable biometric

## Abstract

Human activity recognition (HAR) through gait analysis is a very promising research area for early detection of neurodegenerative diseases because gait abnormalities are typical symptoms of some neurodegenerative diseases, such as early dementia. While working with such biometric data, the performance parameters must be considered along with privacy and security issues. In other words, such biometric data should be processed under specific security and privacy requirements. This work proposes an innovative hybrid protection scheme combining a partially homomorphic encryption scheme and a cancelable biometric technique based on random projection to protect gait features, ensuring patient privacy according to ISO/IEC 24745. The proposed hybrid protection scheme has been implemented along a long short-term memory (LSTM) neural network to realize a secure early dementia diagnosis system. The proposed protection scheme is scalable and implementable with any type of neural network because it is independent of the network’s architecture. The conducted experiments demonstrate that the proposed protection scheme enables a high trade-off between safety and performance. The accuracy degradation is at most 1.20% compared with the early dementia recognition system without the protection scheme. Moreover, security and computational analyses of the proposed scheme have been conducted and reported.

## 1. Introduction

Neurodegenerative diseases are a set of pathologies that develop progressively and irreversibly. These diseases cause a gradual loss of neuronal cells in some central nervous system regions. The factors that determine neurodegenerative diseases are multiple and can be found in genetic, hereditary, and environmental origin [1,2]. The first phase of the disease is difficult to diagnose, as it is subtle. The symptoms become evident when the disease has reached an advanced stage. Moreover, no valid cure allows the patient to recover from the disease; the studies carried out so far have produced results and therapies that can only alleviate the symptoms, delaying the progression [3,4]. For this reason, an early diagnosis is fundamental to delay the evolution of the disease. Some of the most common neurodegenerative diseases are dementia, Parkinson’s, amyotrophic lateral sclerosis, and Huntington’s [5,6,7,8]. HAR through gait analysis is an essential and firmly established tool for early dementia diagnosis, as the significant causes of gait disorders are neurological. Gait analysis is an area of research dedicated to studying human walking, based on analyzing characteristics related to movement, the forces that generate it, and muscle activity [1,2]. Human walking results from complex interactions of components at the nervous system level, and it is greatly influenced by age and behavioral traits related to personality, mood, culture, and society. Regular walking is characterized by a rhythmic, fluid gait, without apparent efforts of joint movement, with freely swinging legs and an upright posture, accompanied by movements of the head, trunk, and arms [9,10]. Recent efforts show the potential of machine learning (ML) approaches for the early detection of neurodegenerative diseases through gait analysis [11,12,13,14], but there are many concerns about the security of these approaches [15,16,17,18]. In healthcare applications, data privacy is a significant challenge. Data should be protected to prevent privacy breaches such as patient identification [19]. Data privacy depends on the characteristics of the data collected and the environment in which they are created and stored [15]. In a healthcare application that uses gait data, the leaked gait data could prevent their use for any future purpose because biometric traits cannot be regenerated [20], or it has been shown that the gait data leak patient’s private attributes [21]. Therefore, the protection of gait data is essential to ensure the patient’s privacy, especially if these data are used for diagnostic purposes. Traditional cryptographic techniques do not provide complete data protection because the data must be decrypted before the classification stage in an ML server. This represents a server-side vulnerability. As a result, homomorphic cryptographic approaches have been developed to perform data processing in the encrypted domain. However, these approaches are subject to multiplicative depth issues and allow a limited number of mathematical operations [22]. For this reason, a neural network architecture must be adapted to perform homomorphic operations. On the other hand, protection techniques have been developed in the biometric context that allows data to be processed in ML algorithms without decryption, such as cancelable biometric approaches. Cancelable biometric approaches ensure biometric data protection through a noninvertible transformation so that the transformed data never revert to their original form. The random projection technique is a cancelable biometric approach that consists of projecting the original feature into a transformed feature matrix through a random projection matrix. The random projection technique ensures the renewability, the noninvertibility of the biometric data, and the system performance, according to the ISO/IEC 24745 security requirements [23]. However, the random projection technique is computationally hard because the number of operations grows exponentially with respect to the size of the matrices involved. Considering the limited resources of the gait feature acquisition device (IoT device), it is necessary to send the patient’s features to a server in a safe manner to perform random projection. The proposed hybrid protection scheme combines homomorphic encryption and the random projection technique to ensure the patient’s gait feature protection in all steps of an early dementia recognition system, from data collection to data classification. In the proposed hybrid protection scheme, the partially homomorphic encryption scheme (PHE) of Paillier is used to ensure security in data transfer so as to perform random projection in the encrypted domain. Subsequently, transformed data are used in the LSTM neural network for early dementia diagnosis. The hybrid protection scheme is independent of the neural network architecture, and thus, it is easily implementable with any neural network. In other words, the proposed hybrid protection scheme combines the advantages of PHE and random projection by solving computational and applicability issues. The main contributions of this work are as follows:

- The proposal of a hybrid protection scheme that combines PHE and a cancelable biometric approach protects the patient’s gait feature and ensures their privacy.
- The adoption of a long short-term memory neural network architecture for the early recognition of dementia, having as input data the multivariate sequences of gait analysis.
- An ablation study on the performance of the proposed protection scheme.
- A comparative analysis between the proposed system and the other state-of-the-art early detection systems for early dementia recognition.
- An evaluation of the security and computational cost of the proposed hybrid protection scheme through security analysis, noninvertibility analysis, renewability analysis, and computational analysis.

The work is organized as follows: Section 2 discusses related work, Section 3 details the proposed hybrid protection scheme, and Section 4 describes the dataset used and explains the conducted experiments to evaluate the system performance and security. Finally, Section 5 discusses the conclusions and possible future developments.

## 2. Related Works

Medical data protection is essential to implement ML systems for early disease detection in real-world contexts.

The patient’s data used in healthcare applications are vulnerable to several attacks that breach the patient’s privacy or compromise the correctness of the diagnosis [17]. Several protection approaches have been proposed based on cryptographic techniques, such as homomorphic encryption, or based on the patient’s anonymization [24,25,26]. Homomorphic encryption is implemented within ML algorithms to perform operations directly on encrypted data to ensure the confidentiality of the data. Several models of secure SVM using homomorphic cryptography are proposed to design secure clinical diagnosis [27,28]. B. Xie et al. [29] propose a secure system for online diagnosis based on multiclass SVM placed on the cloud. In this system, PHE is used for kernel function computation, secure multiplication, and secure comparison to ensure privacy preserving. In [30], an RSA cryptosystem with a homomorphic encryption requirement is used to encrypt medical data before uploading it to the cloud, where the k-nearest neighbor algorithm is applied to perform certain operations on encrypted data, like addition and multiplication. However, homomorphic encryption is applied to shallow learning ML algorithms, and its use in deep learning algorithms presents some limitations. Homomorphic encryption only applies to a limited number of mathematical operations, and thus, a limited number of ML algorithms can directly operate on encrypted data [31]. In addition, multiplicative depth is another problem in implementing a homomorphic cryptography scheme [22]. It is the number of consecutive operations that can be performed on the encrypted data before the noise introduced makes accurate decryption unavailable. The implementation of homomorphic encryption in deep neural networks, such as the LSTM neural network, requires significant changes in the network architecture due to the high multiplicative depth [32]. To solve the issues related to multiplicative depth and the limited number of mathematical operations, the proposed scheme uses homomorphic encryption not directly on the neural network model but to safely perform the random projection technique. Random projection is a cancelable biometric approach developed to protect biometric data. In the case of biometric data, such as human gait, different data protection strategies have been developed compared with traditional cryptographic techniques, such as cancelable biometric approaches. Cancelable biometric approaches use noninvertible transformations to perform intentional and repeatable distortions on the data. In [33], a cancelable biometric approach based on random projection has been used to protect biometric data, achieving accuracy performance similar to the unprotected system. Therefore, biometric data transformed through random projection can be used directly in ML algorithms with limited performance degradation. Data protection with the random projection approach overcomes the multiplicative depth issue in the deep neural network compared with homomorphic encryption. To track human movement and extract kinematic information for medical and health applications, resource-limited devices (cameras, IoT devices) are used [34]. Therefore, random projection cannot be performed directly on these devices. In [35], a hybrid protection scheme combining homomorphic encryption and a cancelable biometric approach was developed. A cancelable biometric approach is applied before the homomorphic encryption to reduce the dimensionality of the data. However, the multiplicative depth issues have not been addressed because the encrypted data have not been used in deep neural networks. In the proposed hybrid protection scheme, homomorphic encryption transforms the data into an encrypted domain to securely perform random projection on the server. In this way, the proposed scheme solves the problem of multiplicative security while maintaining high data security standards. The proposed scheme is being used in conjunction with the LSTM neural network for early dementia recognition in order to evaluate the performance of the scheme in deep neural networks and its applicability in real healthcare contexts.

## 3. Proposed Method

**Figure 1:**
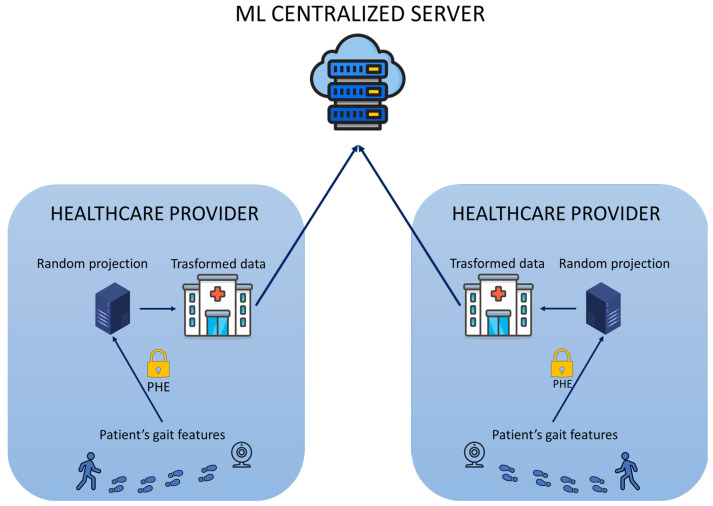
Overall model of an early dementia recognition system with the implementation of the proposed hybrid protection scheme.

Figure 1 depicts the overall model of an early dementia recognition system through gait analysis. Figure 1 shows how the proposed hybrid protection scheme is integrated into the healthcare application. The first part includes the preprocessing of the raw data (video) to obtain the features of the patient’s gait. Coordinate extraction from the videos, sequence creation, Kalman smoothing, and feature extraction were performed to obtain the patient’s gait feature vector. The second phase consists of sending the patient’s data to the healthcare provider server to perform the random projection. The healthcare provider server can potentially be exposed to several attacks or be a secure but nosy server. Patients have no interest in their health data being available to anyone other than their physician. Therefore, the proposed hybrid protection scheme implements the PHE scheme of Paillier to ensure secure communication between the client and the healthcare provider server and secure execution of random projection within the server. After the random projection, the transformed data are stored securely in a centralized database accessible from any hospital, and they can be sent to the ML centralized server without reverting to their original form. There are two possible scenarios: different patients go to different hospitals, or the same patient goes to different hospitals. In both scenarios, the private key is in the exclusive ownership of each hospital department client and is not accessed or shared with other entities. This is fine for the first scenario but could be a problem for the second. However, the result of the decrypted random projection with different private keys will still be the same, starting from the same patient’s data. Hence, the transformed feature vectors of the same patient achieved by different hospitals can be compared for further and different evaluations without the need to share keys among healthcare departments. The following section details the preprocessing and feature extractor phases to understand the biometric data domain to be protected, and subsequently, the proposed hybrid protection scheme is described in detail.

### 3.1. Preprocessing and Feature Extraction

Coordinate extraction is a necessary step in the proposed system intended to extract quantitative information from the movement performed by subjects. Videos are processed frame by frame, extracting the spatial coordinates related to the subjects. More precisely, the image coordinates corresponding to certain parts of each person’s body are estimated. To extract the gait coordinates, the part affinity fields (PAFs) approach was used [4]. It allows for estimating only the key points related to the body parts visible for one or more individuals in the same image. In this work, 14 key points were considered: nose, neck, shoulders (right and left), elbows (right and left), wrists (right and left), hips (right and left), knees (right and left), and ankles (right and left). The extraction process produces for each frame the image related to the frames with the key points “draw” on the owner individuals, the extracted coordinates, and a value of “reliability” of the estimate made. The extracted coordinates were organized into a sequential structure that represents a temporal sequence. More specifically, the sequence of (x,y) coordinates was considered for each key point. From an implementation point of view, the order sequence is as follows: nose, neck, shoulder right, elbow right, wrist right, shoulder left, elbow left, wrist left, hip right, knee right, ankle right, hip left, knee left, and ankle left. While performing this processing step, isolating the coordinates referring to a specific subject from the coordinates of other close individuals is essential. Therefore, a selection mechanism was implemented starting from the initial coordinates referring to a specific subject and iteratively locating the corresponding subject coordinate in the successive frames by calculating the Euclidean distance. The previous phase can occur in some errors. More specifically, there could be some missing coordinates, and the values of consecutive frames could exhibit high oscillations. Therefore, the Kalman filter was used to estimate kinematic values while minimizing the errors [2,36]. A process of peak removal and linear interpolation of missing points was then performed on the coordinates, where a value was assigned to the missing coordinate based on the first previous estimated value and the first subsequent value to generate a data sequence. Features were calculated upon the data sequence just created. These features refer to kinematic information of gait analysis, such as spatiotemporal features and kinematic angles; in addition, sigma–lognormal features were considered [1,2], as reported in Table 1. The sigma–lognormal features are derived from the kinematic theory of rapid human movements [37,38]. This theory can be defined as an instrument to analyze movements as a statistical process that leverages various types of neuromuscular parameters of both the body and the brain. On the basis of this theory, there is the intuition that any movement (movement of wrist, elbow, but also legs, arms, and so on) is the combination of primitives, called strokes, whose velocity and acceleration profile is a lognormal function. A comprehensive description of neurodegenerative disease classification through sigma–lognormal features is provided in [1,2]. At the end of this process, for each user, there are feature vectors referring to the user’s walking directions.

### 3.2. Hybrid Protection Scheme

The feature vectors of each patient are sent to the healthcare provider server to perform random projection. The Paillier PHE scheme was used in the proposed hybrid protection scheme to cipher each feature vector. The Paillier cryptosystem is an asymmetric key scheme that allows a few numbers of computational operations in the cipher domain. Two large prime numbers, *p* and *q*, of equivalent length randomly and independently are used to generate the public and private keys. The public key is defined by (n,g), where n=pq and g=n+1. The private key is defined by (λ,μ), where λ and μ are computed following Equations (1) and (2), respectively:

(1) λ=(p−1)(q−1)

(2) $$\mu \equiv {\left(\left(p-1\right)\left(q-1\right)\right)}^{-1}modn$$

**Figure 2:**
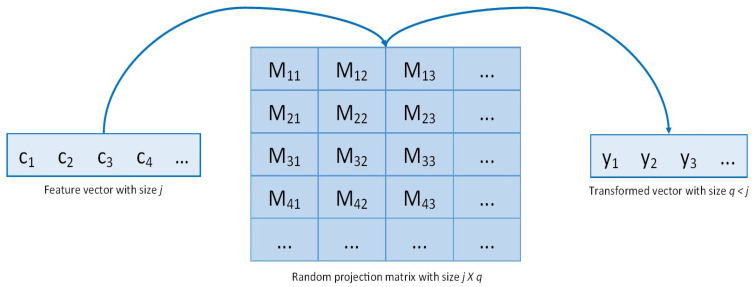
Random projection approach.

The public key is used to cipher the patient’s gait data, and the private key is used to decipher the result of random projection and then to achieve the transformed data. The encryption step is detailed in Equation (3):

(3) $$c\equiv g^{f}\cdot r^{n}\cdot modn^{2}$$

where *c* is the encrypted data and f is the original data to be encrypted. The random projection is performed directly on the encrypted data following Equation (4):

(4) $$y_{cp}=c\cdot M$$

where $y_{c}p$ is the encrypted transformed vector achieved after random projection in the encryption domain, and *c* and *M* are the encrypted feature vector and the random projection matrix, respectively. In the proposed scheme, a Gaussian random matrix was used as a random projection matrix. The Gaussian random matrix is typically used in Gaussian random projection to reduce the data dimensionality, and its components are extracted from the following distribution: $N(0,1/n_{components})$ [39]. The advantage of adopting the Gaussian random matrix is to guarantee a high embedding quality [40]. The random matrix’s column number defines the transformed vector’s size, while the row number is equal to the original feature vector length. Given the matrix *M* of size jXq where j>q, the original feature vector of length *j* is projected into a transformed feature vector of length *q*. A hyperparameter *q* of very small size compared with *j* allows a shorter random projection execution time but impacts the system accuracy that uses the transformed vector as input. In fact, in the random projection, the feature vector is projected into a transformed vector with smaller sizes [41]. This property makes the random projection noninvertible, as detailed in the noninvertibility analysis in Section 4. The Gaussian random matrix is generated through a pseudo-random number generator to ensure the use of the same matrix for each user. A visual representation of the random projection approach is shown in Figure 2. In summary, for each patient, the encrypted gait feature vector is transformed according to Equation (4), and the result is in the cipher domain. Before sending the transformed vector to the ML centralized server, it is necessary to decipher the result following Equation (5). The decryption process does not lead to any security issues because the result is the transformed vector from which the original feature vector can no longer be retrieved.

(5) $$y\equiv L(y_{cp}^{\lambda }\cdot modn^{2})\cdot \mu \cdot modn$$

In Equation (5), *y* represents the transformed vector and *L* is a function defined as L(x)=(x−1)/n. The homomorphic property of the Paillier cryptosystem, as shown in Equation (6), ensures that the result of the decryption process corresponds to the result of the random projection between the original feature vector and the random projection matrix.

(6) $$D({\left(y_{cp}\right)}^{M}modn^{2})=x\cdot M\cdot modn$$

After the decryption process, the transformed feature vector is successively used as input to the LSTM neural network implemented on the ML centralized server for early recognition of dementia by ensuring the patient’s privacy.

## 4. Experimental Results and Analysis

Two case studies were simulated to evaluate the performance of the proposed hybrid protection scheme. In the first case, the system was unprotected, and thus, after the feature extraction, feature vectors were given as input to the LSTM neural network. It represents the baseline system. In the second case, the proposed hybrid protection scheme was implemented, and thus, the feature vectors were given as input to the LSTM neural network in a secure manner. Performance was evaluated based on precision, sensitivity, specificity, F1-measure, accuracy, and AUC ROC. Section 4.1 details the architecture of the LSTM neural network to enable the replicability of the conducted experiments. Further analyses were conducted to evaluate the security and computational cost of the hybrid protection scheme. First, a security analysis was intended to evaluate the security properties of the Paillier PHE scheme. Noninvertibility and renewability analyses were conducted to evaluate the security requirements of the random projection technique according to ISO/IEC 24745. Finally, the execution time of the proposed scheme was computed to evaluate the usability of the proposed scheme in a real context.

### 4.1. LSTM Neural Network Architecture

**Figure 3:**
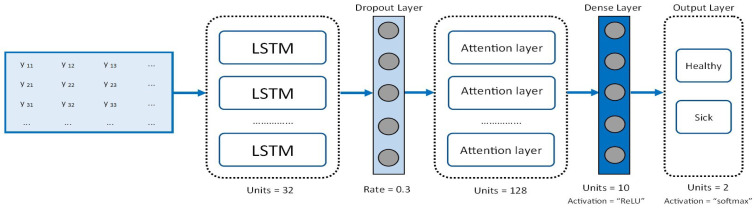
LSTM model architecture.

The LSTM neural network is used for early dementia recognition from transformed patients’ gait data. The choice of implementing the LSTM model is not an essential requirement for the operation of the proposed scheme. The transformed data by the proposed scheme can be used as input in any ML algorithm. The LSTM model is implemented in the centralized ML server without any threat to patients’ privacy because data have been previously protected by the proposed hybrid protection scheme. The neural network architecture consists of an LSTM layer with 32 units, a dropout layer with a dropout rate of 0.3, an attention layer, a dense layer with a ReLU activation function, and a dense output layer with a softmax activation function. The dense layer placed after the attention has a unit number of 10 with L1 regularizer with a value of 0.002 and ReLU activation function; the last layer has two units with a softmax activation function that returns a probability vector. The RMSProp optimizer was used, which implements the RMSProp algorithm, an adaptive optimization method [42]. The binary cross-entropy function was used as the loss function to minimize the classification error for the training examples. In this implementation, the score function was computed as in Equation (7). The LSTM model architecture is illustrated in Figure 3.

(7) $$\mathrm{score}\left(h_{t},h_{s}\right)=h_{t}^{T}\cdot Wh_{s}$$

### 4.2. Dataset

**Figure 4:**
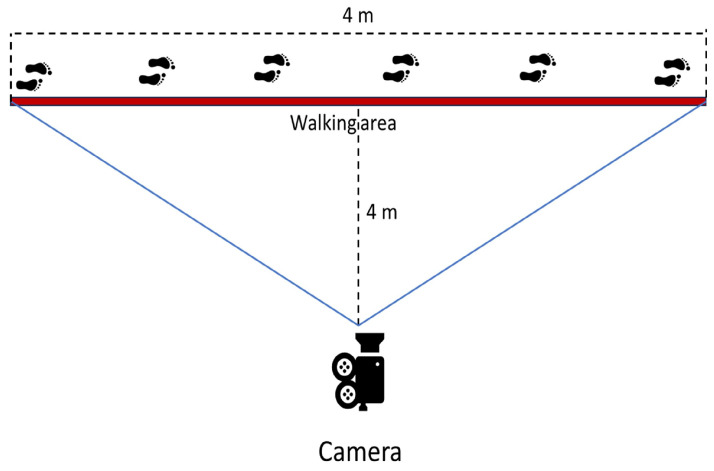
Setup of the data collection process.

**Figure 5:**
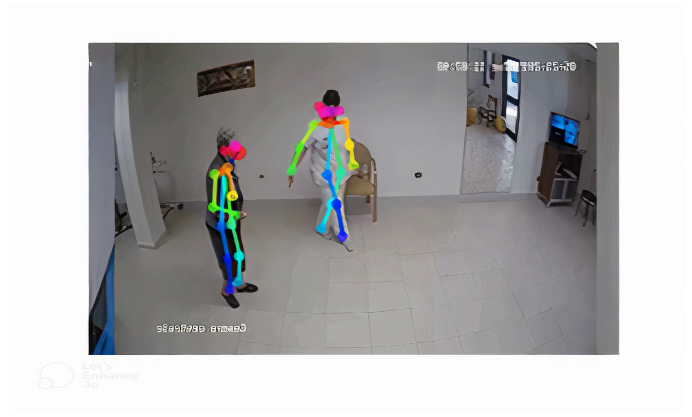
Video capture and preprocessing.

The dataset used in this work includes 118 videos, each of different lengths. The videos were taken at different times and in different hospitals. The dataset includes 43 subjects, of which 23 are healthy controls and 20 patients are affected by a dementia diagnosis by a physician. The dataset contains 2 to 3 videos for each patient. More specifically, 64 videos include healthy control subjects, and 54 videos include patients with dementia. Each subject is filmed laterally, and each subject walks from right to left (or left to right), stops, and retraces the same path in the opposite direction. The inclusion and exclusion criteria of the healthy controls and patients are based on the specific diagnosis, age, stage of the disease, and correct execution of the task, as shown in Table 2. Data collection was performed by a camera placed perpendicularly 4 m from the track to walk. The track to walk is represented by a straight line of 4 m traced on the floor. The setup of the data collection process is shown in Figure 4. Moreover, an actual human image of a subject participating in the video capture and preprocessing processes is shown in Figure 5.

### 4.3. Results

The dataset was divided into training and test sets to evaluate the system performance according to an interpatient separation scheme. More specifically, 14 patients with dementia and 14 healthy subjects were randomly selected to be included in the training set. The remaining individuals were used for the test. Experiments were performed in a 20-fold cross-validation fashion to reduce selection bias and produce more reliable results. A different random matrix was used at each fold to evaluate the impact on performance. This means that the training and testing of the network with this class-balanced interpatient separation scheme is repeated 20 times to obtain 20 different measurements each time with a distinct division of individuals between train and test sets. Table 2 reports the average results obtained for each metric without the proposed protection scheme. Walking directions are considered separately (from left to right and from right to left), and both directions are lumped within a single feature vector in Table 3.

Table 3 shows the best performance obtained when the subject walks from right to left with accuracy and F1-score values of 96.8% and 97.5%, respectively. In the other cases, the accuracy ranges from 96.2% to 96.4% based on the subject walking in both directions and from left to right, respectively. The same experiment was conducted implementing the proposed hybrid protection scheme, and the results are shown in Table 4.

**Figure 6:**
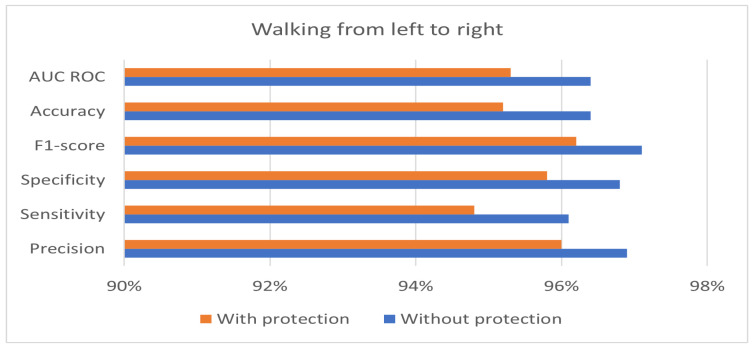
Benchmark of the system accuracy obtained with and without feature protection in the case of walking from left to right.

**Figure 7:**
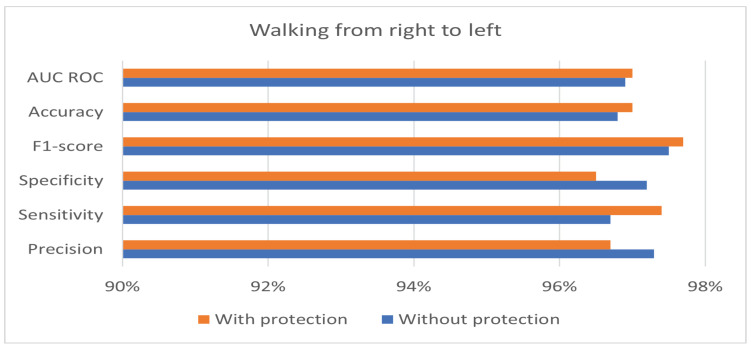
Benchmark of the system accuracy obtained with and without feature protection in the case of walking from right to left.

**Figure 8:**
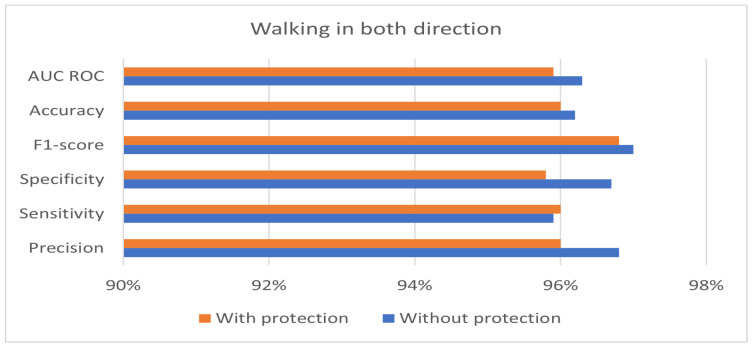
Benchmark of the system accuracy obtained with and without feature protection in the case of walking in both directions.

The private and public keys used in the experiment have a length of 128 bits. Table 4 shows the best performance of the hybrid protection scheme in the case of walking from right to left. Moreover, the results in Table 4 show that the proposed hybrid protection scheme has a very limited impact on system performance compared with the unprotected system. The performance differences between the use of the hybrid protection scheme and without its use are shown in Figure 6, Figure 7 and Figure 8 based on the patient’s walking. Figure 6, Figure 7 and Figure 8 show that the differences in performance are limited between the protected and unprotected systems. The accuracy degradation is at worst 1.20% in the case of walking from left to right. In all other cases, the accuracy difference is under 1%.

In any case, the accuracy results obtained with the proposed hybrid protection scheme range from 94.9% to 97.0% based on the type of walking. The difference (Δ) between the performances in the proposed and unprotected systems is computed and shown in Table 5 for each walking.

Table 5 shows a low performance degradation for each metric. Therefore, there are no evident disadvantages in the performance of implementing the proposed protection scheme. The use of ML methods to predict neurodegenerative disease has several limitations related to privacy and data security that compromise its diffusion in real healthcare contexts. Implementing the proposed scheme represents a solution to encourage ML use in real contexts, overcoming privacy and security issues. It can be implemented in different ML algorithms with limited performance degradation approaches because it is independent of the neural network architecture used to diagnose, as discussed in Section 1.

### 4.4. Comparative Analysis

The performance achieved with the proposed hybrid protection scheme, in the case of walking from right to left, was compared with other state-of-the-art approaches to early dementia diagnosis proposed by V. Dentamaro et al. [2] and M. Cheriet et al. [43]. Systems in [2,43] use the same dataset that was used to evaluate the proposed system but do not implement any approach to protect patients’ features. No state-of-the-art works propose the protection of biometric data used to diagnose neurodegenerative diseases. The proposed system was compared with state-of-the-art works even if no protection techniques were implemented. The comparison is relevant because it shows that the proposed system achieved comparable performance with the state-of-the-art systems, additionally ensuring privacy and data protection. The performance comparison is shown in Table 6.

Table 6 shows that the proposed system achieved performance comparable to other state-of-the-art approaches. The sensitivity, F1-score, accuracy, and AUC ROC obtained by the proposed system are better than those by the systems in [2,43].

### 4.5. Paillier Cryptosystem Security Analysis

The homomorphic nature of the Paillier cryptosystem improves data security related to a traditional cryptosystem. The homomorphic properties enable the execution of computations in the cipher domain without decrypting the data. In other words, the Paillier cryptosystem enables the execution of mathematical operations on the encrypted data and obtains encrypted results without displaying the data in a clear form. The security of the Paillier cryptosystem is based on the composite residuosity class problem. This problem is a mathematical assumption that states that computing *n*-th residue membership classes is computationally hard given moduln and a set of residues moduln [44]. Therefore, recovering the original message from ciphertext is exactly like finding ${\left\|w\right\|}_{g}$. It denotes an integer $x\in Z_{n}$ such that $w=g^{x}\cdot y^{n}\left(modn^{2}\right)$ for some $y\in Z_{n}^{*}$. This problem is denoted as a problem of Class[n]. In the Paillier scheme, n=pq, where *p* and *q* are two large primary numbers used to generate the public and private keys. In the proposed hybrid protection scheme, *p* and *q* with lengths of 64 bits each are used. Therefore, the length of n is 128 bits. Therefore, recovering the original feature vector from the encrypted feature vector is a very hard computational problem. Moreover, the Paillier cryptosystem is robust against cryptanalytic attacks such as the chosen-plaintext attacks. It consists of arbitrarily selecting plaintexts to be encrypted with the cryptographic scheme to obtain information about the secret key. The Paillier cryptosystem has the ciphertext indistinguishability property, and then an attacker cannot obtain any information from the ciphertexts. Finally, the homomorphic property of the Paillier cryptosystem allows for performing operations on the encrypted data, further improving security.

### 4.6. Noninvertibility Analysis

The security of the random projection was evaluated according to the standard ISO/IEC 24745 in terms of noninvertibility and renewability of the transformation used. If a transformation is noninvertible, an attacker cannot retrieve the original data from the transformed data. The noninvertibility of the random projection is ensured by the Rouché–Capelli theorem. The theorem affirms that in a linear system, there are infinite solutions if the linear equation system is non-full-rank [45]. In the proposed hybrid protection scheme, the linear equation system generated from the transformed feature vector and the random projection matrix is non-full-rank. Therefore, an attacker cannot retrieve the original feature from this information. In the proposed scheme, the column number of the projection matrix is smaller than the length of the original feature vector, as detailed in Section 3.2. Therefore, an attacker that knows the transformed feature vector and the random projection matrix should solve a linear system where the number of variables (values of the original feature vector) is larger than the number of equations to obtain the values of the original feature vector. For example, given a transformed feature vector $y=[y_{1},y_{2},y_{3}]$ and the random matrix *M* of size jq,

$$M=\left[\begin{array}{ccc} m_{11} & m_{12} & m_{13}\\ m_{21} & m_{22} & m_{23}\\ m_{31} & m_{32} & m_{33}\\ m_{41} & m_{42} & m_{43}\\ m_{51} & m_{52} & m_{53} \end{array}\right]$$

The original feature vector $x=[x_{1},x_{2},x_{3},x_{4},x_{5}]$ of length *j* is obtained by solving the following linear system:

$$\begin{array}{c} m_{1,1}x_{1}+m_{2,1}x_{2}+m_{3,1}x_{3}+m_{4,1}x_{4}+m_{5,1}x_{5}=y_{1}\\ m_{1,2}x_{1}+m_{2,2}x_{2}+m_{3,2}x_{3}+m_{4,2}x_{4}+m_{5,2}x_{5}=y_{2}\\ m_{1,3}x_{1}+m_{2,3}x_{2}+m_{3,3}x_{3}+m_{4,3}x_{4}+m_{5,3}x_{5}=y_{3} \end{array}$$

The linear system can be represented in matrix form

$$\left(M^{T}|y\right)$$

where $M^{T}$ is the transpose of *M* that represents the coefficient matrix. The solutions of the linear system are infinite because the rank $\left(M^{T}|y\right)$ is smaller than the number of variables $x_{i}$. Therefore, if the random matrix size fulfills the requirement that q<j, an adversary cannot obtain the original features from the transformed features.

### 4.7. Renewability Analysis

The renewability property is to renew biometric data if they are compromised or for essential security needs. The original biometric data cannot be renewed because they are unique, but a protected biometric template can be renewed simply by changing the parameters used for transformation. It is an essential characteristic of the biometric cancelable approaches. In the proposed hybrid protection scheme, the renewability requirement of the protected biometric data is ensured by changing the seed of the pseudo-random number generator used to generate the random projection matrix. Therefore, different random projection matrices can be generated using different seeds. Different matrices make it possible to compute different transformed feature vectors from the same original feature. In other words, different transformed gait feature vectors can be generated from the same users to retrain the system in case of data breaches. Therefore, the attacker cannot use the compromised data to diagnose users’ diseases.

### 4.8. Computational Analysis

The running time of the proposed hybrid protection scheme was computed to evaluate the computational cost. Table 7 shows the execution time of the encryption phase of the gait feature vector, the random projection, and the decryption phase of the results in seconds. The execution time was computed by using a feature vector of size j=30 and setting a size of transformed vector (*q*) of j−1. A larger size of the feature vector and hyperparameter *q* impact the execution time. The experiment was conducted on a PC with CPU AMD Ryzen Threadripper 1920X 12-Core, RAM 64 Gb, and GPU Nvidia Titan RTX with 24 Gb.

Table 7 shows the total running time to obtain a protected patient’s gait feature of 57.259 s. The encryption and decryption phases are performed by the healthcare provider client, while the healthcare provider server performs the random projection phase. The computational cost of the protection scheme for the client is 3.3609 s. Therefore, the client is always within the availability of the healthcare provider, and the proposed protection approach has very minimal impact on the flow of hospital activities. Furthermore, the running time to assess the presence of dementia in a patient was computed. In this case, the patient’s gait data are sent in a secure way to the ML server, where the LSTM model evaluates the presence of dementia and sends the result to the client. The average time to evaluate the disease is 0.002 s for each patient.

## 5. Conclusions

A hybrid protection scheme to protect patients’ gait features in a health system for early dementia detection has been proposed. The proposed hybrid protection scheme uses the PHE Paillier cryptosystem and the random projection approach to protect the patient’s gait feature. The proposed system has a minimal impact on early detection performance. The best absolute accuracies were reached on walking from right to left. The comparative analysis shows that the proposed system achieved better performance in terms of sensitivity, F1-score, accuracy, and AUC ROC than the other state-of-the-art systems without data protection. Moreover, the security analysis shows the robustness of the Paillier cryptosystem against brute-force attacks and chosen-plaintext attacks. The noninvertibility and renewability analyses show the security requirements of the random projection technique according to ISO/IEC 24745. The proposed hybrid protection scheme is independent of the ML approaches used because it does not require any modification of the neural network architecture. Therefore, the proposed hybrid protection scheme can be implemented with other ML algorithms to improve early dementia recognition performance. A limitation of the proposed scheme is related to the efficiency of the homomorphic encryption scheme used. The execution time of the Paillier cryptosystem in encryption, computation, and decryption depends on the size of the original and transformed feature vector. It can grow exponentially as the size increases. In the future, the Paillier cryptosystem could be replaced with more efficient homomorphic encryption schemes, such as CKKS, in the proposed scheme. Moreover, the proposed scheme will be tested with other state-of-the-art models of neural networks and with other human gait datasets for neurodegenerative disease prediction to further validate the proposed scheme’s results. Secure private key storage and key length represent the challenges of the proposed scheme. Compromising the private key would allow an unauthorized user to decrypt the patient’s data. On the other hand, a longer key length inevitably leads to an increase in the execution time of the proposed protection scheme. The hybrid protection scheme can be used to protect different biometric traits used in different healthcare applications (such as speech to predict Parkinson’s disease). The proposed scheme can be easily adapted to protect different biometric features compared with human gait.

## Figures and Tables

**Table 1:** Extracted features.

Category	Feature	Description
Temporal space	Displacement	$d_{i}=\sqrt{\Delta x_{i}^{2}+\Delta y_{i}^{2}}$
	Displacement x	$\Delta x_{i}=x_{i+1}-x_{i}$
	Displacement y	$\Delta y_{i}=y_{i+1}-y_{i}$
	Velocity	$v_{i}=\frac{d_{i}}{\Delta t_{i}}$
	Velocity x	$v_{x,i}=\frac{\Delta x_{i}}{\Delta t_{i}}$
	Velocity y	$v_{y,i}=\frac{\Delta y_{i}}{\Delta t_{i}}$
	Acceleration	$a=\frac{v_{i}}{\Delta t_{i}}$
	Acceleration x	$a_{x,i}=\frac{v_{x,i}}{\Delta t_{i}}$
	Acceleration y	$a_{y,i}=\frac{v_{y,i}}{\Delta t_{i}}$
	Tangent angle	$\rho_{i}=\tan^{-1}\left(\frac{\Delta y_{i}}{\Delta x_{i}}\right)$
Sigma–lognormal features	Lognormal stroke number	Number of lognormal strokes
	*D* parameter	*D* parameter for all lognormal strokes
	μ parameter	μ parameter for all lognormal strokes
	σ parameter	σ parameter for all lognormal strokes
	θ parameter	θ parameter for all lognormal strokes
Corners	Nose–neck–hip	Angle between nose, neck, and hip
	Neck–hip–knee	Angle between neck, hip, and knee
	Shoulder–elbow–wrist	Angle between shoulder, elbow, and wrist
	Hip–knee–ankle	Angle between hip, knee, and ankle
	Right knee–hip–left knee	Angle between right knee, hip, and left knee

**Table 2:** Inclusion and exclusion criteria.

Inclusion Criteria	Exclusion Criteria
**Patients**	
Adults aged 65 to 90 years	Refusal to give informed consent
Diagnosis of mild to severe dementia	Any condition that would limit the ability of the patient to participate in the study
Gender-inclusive: 6 men and 14 women	Patients who did not complete all the required walking tasks
**Healthy controls**	
Adults aged 30 to 75 years	Refusal to give informed consent
No dementia diagnosis	Subject who did not complete all the required walking tasks

**Table 3:** Results without the proposed hybrid protection scheme.

Score	Walking from Left to Right	Walking from Right to Left	Walking in Both Directions
Precision	96.9%	**97.3%**	96.8%
Sensitivity	96.1%	**96.7%**	95.9%
Specificity	96.8%	**97.2%**	96.7%
F1-score	97.1%	**97.5%**	97.0%
Accuracy	96.4%	**96.8%**	96.2%
AUC ROC	96.4%	**96.9%**	96.3%

**Table 4:** Results with the proposed hybrid protection scheme.

Score	Walking from Left to Right	Walking from Right to Left	Walking in Both Directions
Precision	96.0%	**96.7%**	96.0%
Sensitivity	94.8%	**97.4%**	96.0%
Specificity	95.8%	**96.5%**	95.8%
F1-score	96.2%	**97.7%**	96.8%
Accuracy	95.2%	**97.0%**	96.0%
AUC ROC	95.3%	**97.0%**	95.9%

**Table 5:** Differences in performance between the proposed and unprotected systems.

Score	Walking from Left to Right	Walking from Right to Left	Walking in Both Directions
Δ Precision	0.9%	0.6%	0.8%
Δ Sensitivity	1.3%	−0.7%	−0.1%
Δ Specificity	1.0%	0.7%	0.9%
Δ F1-score	0.9%	−0.2%	0.2%
Δ Accuracy	1.2%	−0.2%	0.2%
Δ AUC ROC	1.1%	−0.1%	0.4%

**Table 6:** Comparative analysis between state-of-the-art systems and the proposed system.

Work	Prec.	Sens.	Spec.	F1	Acc.	AUC
[2]	**97.7%**	96.9%	**97.1%**	96.7%	96.9%	96.9%
[43]	95.9%	95.3%	95.7%	95.5%	95.5%	96.1%
Proposed system	96.7%	**97.4%**	96.5%	**97.7%**	**97.0%**	**97.0%**

**Table 7:** Running time of the proposed hybrid protection scheme.

Phase	Running Time (s)
Encryption	3.322
Random projection	53.899
Decryption	0.0389
Total	57.259

## Data Availability

The dataset is provided anonymously and does not contain videos of people’s faces.
